# Septic cardiomyopathy

**DOI:** 10.1186/2110-5820-1-6

**Published:** 2011-04-13

**Authors:** Antoine Vieillard-Baron

**Affiliations:** 1 Service de Réanimation, Hôpital Ambroise Paré, Assistance Publique des Hôpitaux de Paris, 9 avenue Charles de Gaulle, 92104 Boulogne, France; 2 Faculté de Médecine de Paris Ile de France Ouest, Université de Versailles Saint Quentin en Yvelines, 78000 Versailles, France

## Abstract

Depression of left ventricular (LV) intrinsic contractility is constant in patients with septic shock. Because most parameters of cardiac function are strongly dependent on afterload, especially in this context, the cardiac performance evaluated at the bedside reflects intrinsic contractility, but also the degree of vasoplegia. Recent advances in echocardiography have allowed better characterization of septic cardiomyopathy. It is always reversible providing the patient's recovery. Unlike classic cardiomyopathy, it is not associated with high filling pressures, for two reasons: improvement in LV compliance and associated right ventricular dysfunction. Although, it is unclear to which extent it affects prognosis, a hyperkinetic state is indicative of a profound and persistent vasoplegia associated with a high mortality rate. Preliminary data suggest that the hemodynamic response to a dobutamine challenge has a prognostic value, but large studies are required to establish whether inotropic drugs should be used to treat this septic cardiac dysfunction.

## Introduction

Reversible myocardial depression in patients with septic shock was first described in 1984 by Parker et al. using radionuclide cineangiography [1]. In a series of 20 patients, they reported a 65% incidence of left ventricular (LV) systolic dysfunction, defined by an ejection fraction <45% [1]. In 1990, using transthoracic echocardiography, Jardin et al. reported the same results [2]. In a canine model simulating human septic shock, Natanson et al. demonstrated that intrinsic LV performance was actually depressed in all animals and not corrected by volume expansion [3]. Finally, more recently, Barraud et al. confirmed the presence of severe depressed intrinsic LV contractility using LV pressure/volume loops in lipopolysaccharide-treated rabbits [4]. All of these studies, and many others not cited in this introduction, demonstrate the reality of the impairment of intrinsic LV contractility in septic shock. For many years, septic cardiac dysfunction was largely underestimated because the hemodynamic device used, i.e., the pulmonary artery catheter, was not appropriate for establishing such a diagnosis. Development of new hemodynamic tools at the bedside, such as echocardiography, allowed better characterization of the septic cardiomyopathy [5]. The following review explains the mechanisms of such a depression, its characteristics, incidence, and finally its impact on treatment and prognosis. We decided not to deal with the place and the role of biomarkers, which will be presented in a future review of the journal.

### Mechanisms

Many factors may contribute to cardiac depression during sepsis. Studies performed in humans have ruled out coronary hypoperfusion requiring coronary intervention as a cause of LV systolic dysfunction in sepsis [6,7]. Of course, patients with coronary disease may behave differently.

On the other hand, the role of cytokines has been strongly advocated in the genesis of septic cardiomyopathy. In 1985, Parrillo et al. demonstrated in vitro that myocardial cell shortening is reduced by exposure to the serum of septic patients [8]. Later, the same team showed that the circulating factor responsible for this was tumor necrosis factor α (TNF-α) [9,10], even though later studies have implicated other cytokines, such as interleukin-1β [11]. Kumar et al. suggested that the effect of cytokines on cardiac myocytes results from an increase in intracellular cGMP and in nitric oxide [12]. In addition, direct alteration in cellular respiration with mitochondrial dysfunction also was advocated [13], and, finally, Tavernier et al. suggested that increased phosphorylation of troponin I was involved by reducing myofilament response to Ca2+ [14].

### Main characteristics of septic cardiomyopathy

The first characteristic of septic cardiomyopathy is that it is acute and reversible, providing the patient recovers. In 90 patients during a 5-year period, Jardin et al. reported that LV ejection fraction is normalized in a few days [15], as also reported more recently by Bouhemad et al. [16].

**Figure 1:**
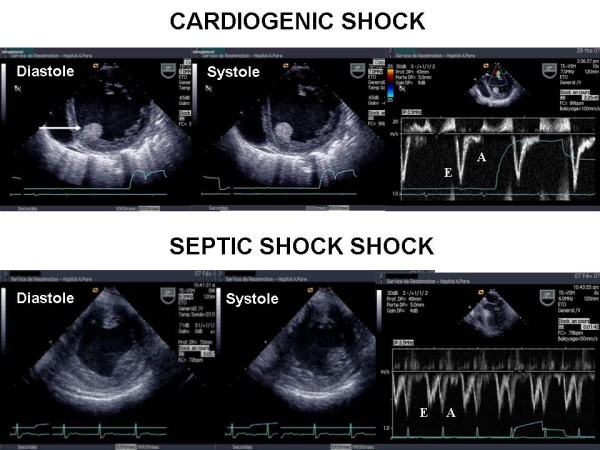
**Transesophageal echocardiography in two patients--one with cardiogenic shock (above) and the other with septic shock (below)**. In the patient with cardiogenic shock, the left ventricular short-axis view demonstrated global hypokinesia of the left ventricle with major dilatation. Pulsed Doppler at the mitral valve demonstrated a restrictive pattern of the left ventricular inflow with a high E wave velocity and a very low A wave velocity, highly suggestive of a high LV filling pressure. Note also the thrombus in the left ventricle (arrow). In the patient with septic shock, the short-axis view also demonstrated global hypokinesia of the left ventricle, but without a major dilatation. Note also the Doppler profile at the mitral valve, highly suggestive of normal LV filling pressure.

The second characteristic, which is crucial to full understanding, is that depressed LV systolic function is associated with normal or low LV filling pressure, unlike the "classic" pattern of cardiogenic shock where LV pressures are elevated (Figure 1). This may explain why the pulmonary artery catheter has for many years underestimated the incidence of LV systolic dysfunction. Jardin et al. and Bouhemad et al. reported an average pulmonary capillary wedge pressure close to 11 mmHg in patients with decreased LV ejection fraction, which is not significantly different from that found in patients with a preserved ejection fraction [2,16]. In the study by Parker et al., the pulmonary capillary wedge pressure was 14 mmHg on average in patients with LV ejection fraction <45% [1].

**Figure 2:**
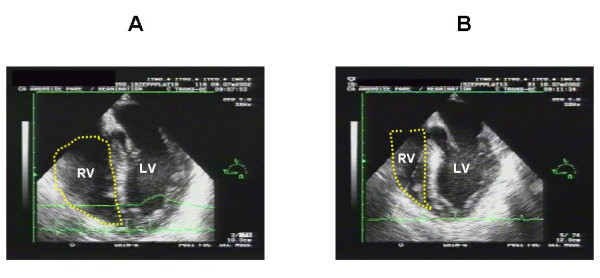
**Long-axis view of the left ventricle by a transesophageal approach in a patient ventilated for septic shock**. At day 1 (panel A), the patient had right ventricular dysfunction illustrated by major dilatation of the right ventricle. At day 2 (panel B), this was corrected. RV, right ventricle; LV, left ventricle.

Two mechanisms may explain this absence of elevated LV pressures. The first relates to the frequent association with right ventricular (RV) dysfunction. Vincent et al. in a group of 93 patients with septic shock reported a decreased RV ejection fraction compared with a "control" group [17]. Similar results were found by Kimchi et al. and Parker et al. [18,19]. Using transesophageal echocardiography, we reported that almost 30% of patients have RV dilatation, which is highly suggestive of significant RV dysfunction (Figure 2) [20]. RV dysfunction is related to acute pulmonary hypertension, which is frequently associated in this situation because of the acute lung injury, or depressed intrinsic contractility due to circulating cytokines [18]. It protects the pulmonary circulation [21] and avoids significant elevation of LV pressures.

The second mechanism relates to LV compliance alteration, which usually occurs. In their original work, Parker et al. suggested a huge increase in LV compliance; they found a dilatation of the left ventricle of more than 100% [1]. This very impressive LV "preload adaptation" was actually never confirmed and was probably explained in part by technical errors related to the use of the pulmonary artery catheter. Most studies using echocardiography only report a slight increase in LV size in patients with decreased LV ejection fraction compared with patients with preserved ejection fraction, suggesting a true but slight increase in LV compliance in these patients (Table 1) [16,20,22]. In 12 normal healthy volunteers, Suffredini et al. demonstrated that injection of endotoxin induces a depression of LV systolic function associated with a significant decrease in the ratio of pulmonary capillary wedge pressure to LV end-diastolic volume index [23]. A limited but significant increase in LV end-diastolic volume (+15%) was reported after volume loading with a pulmonary capillary wedge pressure less augmented than in the control group [23].

**Table 1:** End-diastolic size of the left ventricle according to the ejection fraction

	LV end-diastolic size	
	**Decreased LVEF**	**Preserved LVEF**
	
Parker et al. [1] 20 patients, *PAC*	*LVEDV *159 ± 29 mL/m^2^	81 ± 9 mL/m^2 ^*
Jardin et al. [2] 21 patients, *TTE*	*LVEDV *76 ± 18 mL/m^2^	70 ± 20 mL/m^2^
Jardin et al. [15] 90 patients, *TTE*	*LVEDV *80 ± 21 mL/m^2^	62 ± 15 mL/m^2 ^*
Vieillard-Baron et al. [25] 67 patients, *TEE*	*LVEDV *76 ± 24 mL/m^2^	68 ± 24 mL/m^2^
Bouhemad et al. [16] 45 patients, *TEE*	*LVEDA *13 ± 3 cm^2^/m^2^	11 ± 2 cm^2^/m^2 ^*

PAC, pulmonary artery catheter; TTE, transthoracic echocardiography; TEE, transesophageal echocardiography; LVEF, left ventricular ejection fraction; LVEDV, left ventricular end-diastolic volume; LVEDA, left ventricular end-diastolic area.

*p < 0.05.

### Incidence

**Figure 3:**
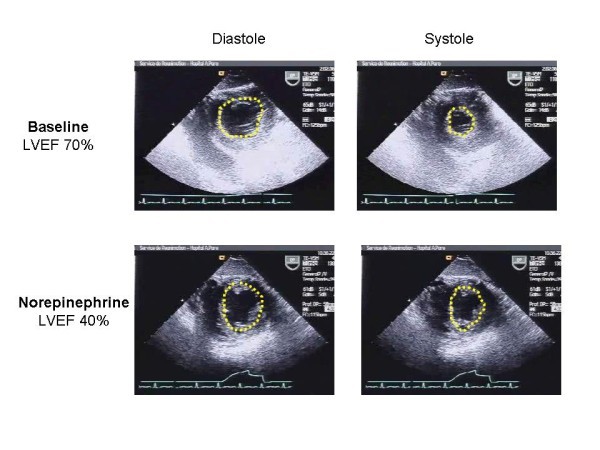
**Short-axis view of the left ventricle by a transgastric approach in a patient with septic shock at baseline after initial resuscitation and after a few hours of norepinephrine infusion**. Note that restoration of a "normal" left ventricular afterload has unmasked impaired contractility. LVEF, left ventricular ejection fraction.

**Figure 4:**
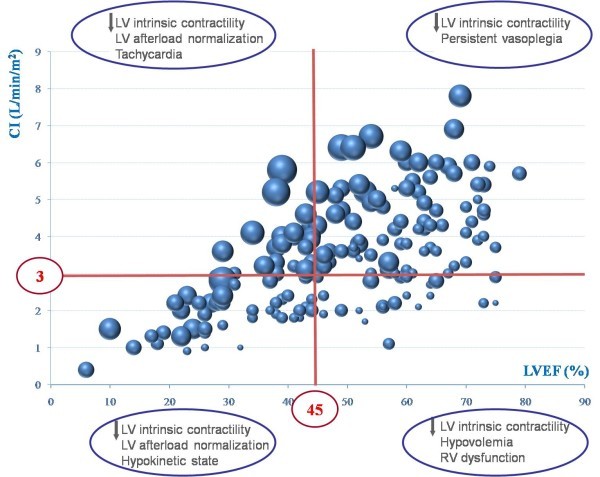
**Relationship between left ventricular (LV) ejection fraction (x axis) and cardiac index (y axis) in 183 patients with septic shock who underwent echocardiography**. Providing that depressed LV intrinsic contractility is constant, the relation may be separated into four parts according to systemic vascular resistance, volemia, and right ventricular (RV) function. Size of the circles is related to LV end-diastolic volume, from 35 for the smallest to 135 ml for the biggest.

The reported incidence of LV systolic dysfunction varies significantly (Table 2). This is easy to understand, because the LV afterload plays a crucial role in the evaluation of cardiac function. As well illustrated by Robotham et al. [24], the same value of LV ejection fraction may correspond to very different levels of intrinsic LV contractility: for instance, an ejection fraction of 60% may correspond to a severe impairment of LV contractility, if the afterload is very low, as it is in septic shock before resuscitation. As shown by all animal studies, we must consider that the presence of depressed LV intrinsic performance is constant in septic shock. Accordingly, LV ejection fraction actually reflects the LV afterload rather than the intrinsic contractility. During the 6 first hours of resuscitation, we found an 18% incidence of a hypokinetic profile, associating low cardiac index and decreased LV ejection fraction [20]. In another study, where echocardiography was performed later, we reported a 40% incidence of LV systolic dysfunction after 24 hours of resuscitation [25]. In the same study, the incidence increased to 60% after 2 and 3 days [25], probably because of the restoration of a normal LV afterload by norepinephrine or by the natural evolution of the infectious process. This is why echocardiography, when repeated at different times, may show how norepinephrine infusion may unmask poor intrinsic LV contractility (Figure 3). Interestingly, in the study by Jardin et al., the systemic vascular resistances were significantly lower in patients with a preserved LV ejection fraction than in patients with decreased ejection fraction [2]. A similar result was reported by Parker et al. [1]. In our study, 15% of patients after 6 hours had a hyperkinetic profile, associating tachycardia, supra-normal LV ejection fraction, small LV cavity despite massive volume expansion, and high cardiac index--a pattern reflecting persistent and very profound vasoplegia [20]. In 2003, we reported a series of 183 patients with septic shock who were all monitored hemodynamically by using echocardiography through transthoracic or transesophageal route [5]. As shown in Figure 4, the relationship between LV ejection fraction and cardiac index may be separated into four parts, depending on LV afterload, volemia, and RV function.

**Table 2:** Incidence of LV systolic dysfunction in septic shock according to the time of evaluation

	Time of study/admission	Incidence of LV systolic dysfunction
Parker et al. [1] *PAC + radionuclide cineangiography*	Day 1	65%
Jardin et al. [2] *TTE*	0-6 hours	29%
Vieillard-Baron et al. [20] *TEE*	0-6 hours	18%
Vieillard-Baron et al. [25] *TEE*	Day 1, 2, 3	60%
Bouhemad et al. [16] *TEE*	?	20%
Etchecopar-Chevreuil et al. [22] *TEE*	12 hours	46%

PAC, pulmonary artery catheter; TTE, transthoracic echocardiography; TEE, transesophageal echocardiography.

### Prognosis and treatment

It is very difficult to establish whether septic cardiomyopathy independently affects the prognosis of patients with septic shock, because many other variables are involved, such as age, patient history, type of microorganisms, and time to resuscitation. Initially, Parker et al. suggested that development of septic cardiomyopathy was "protective" [1]. Ten of the 13 survivors had an LV ejection fraction <40%, but none of the nonsurvivors [1]. In appearance, we found different results with a mortality rate of 43% in patients with a hypokinetic profile compared with 24% in patients with a normokinetic profile [20]. We also found that patients with a hyperkinetic profile (small left ventricle, supranormal ejection fraction, tachycardia, high cardiac index) had a 100% mortality rate [20]. Actually, rather than a "protective" effect of LV systolic dysfunction, we can conclude that the prognosis is poor in the presence of a hyperkinetic state, which reflects persistent and profound vasoplegia, as explained above. Table 3 summarizes the main studies and their results in terms of survival and LV systolic function.

**Table 3:** LV ejection fraction and cardiac index in the survivors and non-survivors of septic shock

	Survivors (n = 99)	Nonsurvivors (n = 101)
Parker et al. [1] *20 patients*	*LVEF *32 ± 4% *CI *4.1 ± 0.4 L/min/m^2^	55 ± 3 5.4 ± 0.7 L/min/m^2^
Jardin et al. [15] *90 patients*	*LVEF *44 ± 16% *CI *3.6 ± 0.3 L/min/m^2^	52 ± 14% 3.7 ± 0.4 L/min/m^2^
Vieillard-Baron et al. [25] *67 patients*	*LVEF *49 ± 18% *CI *3.1 ± 0.9 L/min/m^2^	55 ± 15% 3.8 ± 1.3 L/min/m^2^
Kumar et al. [32] *23 patients*	*LVEF *50 ± 5% *CI *2.78 ± 0.3 L/min/m^2^	57 ± 4% 3.01 ± 0.3 L/min/m^2^

LVEF, left ventricular ejection fraction; CI, cardiac index.

Three therapeutic interventions must be discussed. The first is volume expansion. Parker et al. suggested that "massive" fluid infusion maintains a normal cardiac index, despite a significantly impaired LV contractility, through LV preload adaptation [1]. However, we have learned that this dilatation is limited and that fluid overload is deleterious for survival [26,27]. In the study by Ognibene et al., volume infusion was unable to restore normal left ventricular function and unmasked a flat Frank-Starling curve in the 21 patients with septic shock [28]. In our study, LV stroke index and LV end-diastolic volume did not correlate, whereas LV stroke index and LV ejection fraction were strongly correlated [20]. Nevertheless, volume expansion must always be proposed first to restore central blood volume in the case of absolute or relative hypovolemia.

The second therapeutic option is the infusion of an inotropic drug, such as dobutamine, a beta-agonist agent. We and others reserve this treatment for patients with persistent shock, lactic acidosis, and oliguria [25,29]. In this situation, dobutamine increases LV ejection fraction and cardiac index [25,29]. To which extent such a treatment may improve the patient's prognosis is unknown. However, it is well established that the cardiovascular response to dobutamine stress predicts outcome in sepsis. Vallet et al. using a dose of 10 μg/kg per min and Rhodes et al. using a dose of 5 μg/kg per min reported that patients with an increased oxygen consumption (>15%) in response to dobutamine infusion have a much higher survival rate [30,31]. This was related to a significant increase in cardiac index and oxygen delivery [30,31]. More recently, Kumar et al. performed a "dobutamine challenge" at 5, 10, and 15 μg/kg per min in 23 patients with septic shock [32]. They also found that survival was associated with increased cardiac performance and LV contractility indices [32]. In particular, a cutoff value of 8.5 mL/m^2 ^increase in LV stroke index in response to dobutamine correctly categorized the outcome in 21 of 23 patients [32]. Levosimendan, a new calcium sensitizer, also has been proposed to treat septic cardiomyopathy. It may improve not only LV but also RV function in the context of sepsis [33,34]. Barraud et al. in an endotoxin model in rabbits reported that LV systolic elastance was restored during levosimendan infusion [4].

Finally, a few words should be mentioned about norepinephrine administration. As explained above and shown in Figure 3, norepinephrine infusion may unmask the impairment of LV contractility. However, some authors recently have suggested that administration of norepinephrine for restoring mean arterial pressure in the early phase of septic shock also increased cardiac output through an increase in both cardiac preload and cardiac contractility [35]. Such findings remain to be confirmed.

## Conclusions

Depression of LV intrinsic contractility is constant in patients with septic shock. Because most parameters of cardiac function are strongly dependent on afterload, especially in this context, cardiac performance evaluated at the bedside reflects intrinsic contractility but also the degree of vasoplegia. Recent advances in echocardiography have allowed better characterization of septic cardiomyopathy. Unlike classic cardiomyopathy, it is not associated with high filling pressures for two reasons: increased LV compliance and frequently associated RV dysfunction. It is always reversible. Although it is unclear how septic cardiomyopathy affects outcome, a hyperkinetic state is indicative of profound and persistent vasoplegia associated with a high mortality rate. Preliminary data suggest that the hemodynamic response to dobutamine challenge has a prognostic value, but large studies are required to establish whether inotropic drugs should be used to treat this septic cardiac dysfunction.

## Competing interests

The author declares that they have no competing interests.
